# Fabrication of 512-Channel Geometrical Passive Breakup Device for High-Throughput Microdroplet Production

**DOI:** 10.3390/mi10100709

**Published:** 2019-10-18

**Authors:** Chul Min Kim, Gyu Man Kim

**Affiliations:** 1 School of Mechanical Engineering, Kyungpook National University, Daegu 41566, Korea; faithfulsaint@daum.net; 2 Department of Mechanical Engineering, Korea Polytechnic University, Siheung-Si, Gyeonggi-Do 15073, Korea

**Keywords:** microsphere, high throughput, droplet splitting, microfluidics, T-junction

## Abstract

We present a 512-microchannel geometrical passive breakup device for the mass production of microdroplets. The mass production is achieved through the passive breakup of a droplet into two droplets. The microchannel geometry in the microfluidic device was designed and optimized by focusing on stable droplet splitting for microdroplet preparation and minimizing the hydraulic resistance of the microchannel for achieving high throughput; the minimization of hydraulic resistance was achieved by employing analytical approaches. A total of 512 microdroplets could be prepared from a single liquid plug by making the liquid plug pass through nine sequential T-junctions in the microfluidic device, which led to the splitting of droplets. The microfluidic device was fabricated using conventional photolithography and polydimethylsiloxane (PDMS) casting. We estimated the performance of the microfluidic device in terms of the size distribution and production rate of microdroplets. Microdroplets with a diameter of 40.0 ± 2.2 µm were prepared with a narrow size distribution (coefficient of variation (CV) < 5.5%) for flow rates of disperse (*Q*_d_) and continuous phase (*Q*_c_) of 2 and 3 mL/h, respectively. Microdroplet production rates were measured using a high-speed camera. Furthermore, monodisperse microdroplets were prepared at 42.7 kHz for *Q*_d_ and *Q*_c_ of 7 and 15 mL/h, respectively. Finally, the feasibility of the fabricated microfluidic device was verified by using it to prepare biodegradable chitosan microspheres.

## 1. Introduction

Microspheres, also known as microparticles, have a spherical microstructure with a diameter in the range of 1–1000 μm [1]. They are used to deliver high-cost functional materials such as cells, protein drugs, and nanomaterials by entrapping the materials in a polymer matrix or attaching them to the microsphere surface [2,3,4]. Because the microsphere size distribution is a major factor influencing the controlled release of functional materials [5], the preparation of monodispersed microspheres is getting more important. However, conventional preparation methods such as spray drying, solvent evaporation, and coacervation, have limitations with regard to the preparation of monodispersed microspheres [2]. The polydispersed microspheres from these conventional preparation methods may cause undesirable effects in terms of the degradation rate of microparticle, the stability of the loaded functional materials, and the functional material release property [6].

For decades, microfluidic systems were considered as a promising research tool in various fields such as chemistry, biology, and medicine because of their portability, multifunction integration potential, and production capability of picoliter- to nanoliter-sized droplets [7,8,9].

Droplets can be used to produce monodispersed microdroplets through microfluidics, accurately controlling the loading of functional materials [3]. Moreover, it can be efficiently integrated with another system for multipurpose applications [10]. However, microfluidic-system-based droplet production has a limitation: it can be scaled up only to a limited extent, because a serial process is used to produce microspheres on a nanoliter scale [11]. Various researches were conducted to overcome this limitation, including the use of a robust device, parallel inlets, and multiple nozzles. However, the resulting preparation methods were complex and showed low repeatability [12,13,14].

The droplet splitting approach can provide a solution to the aforementioned limitation [15]. There are several studies on droplet splitting at T-junctions in microchannels [11,15]. A single microdroplet is split into two at a T-junction, and this feature can be employed for preparing monodisperse microdroplets [11,15,16]. The number of microdroplets prepared depends on the number of T-junctions. Therefore, it is possible to produce microdroplets as a function of the number of T-junctions.

We previously reported the first application of a geometrical passive breakup T-junction microfluidic device for preparation of biocompatible microspheres [11]. However, although monodisperse microdroplets could be prepared at 9 kHz, it was necessary to increase the microdroplet production rate for achieving high throughput. Furthermore, there was no study on the design of microchannels for minimizing flow resistance to increase the flow rate.

In this paper, we present a 512-channel geometrical passive breakup device that is capable of large-scale production of monodisperse microdroplets. The microchannel was designed on the basis of microfluidic analytics for optimizing the channel geometry and minimizing the hydraulic resistance. The microfluidic device was fabricated using conventional photolithography and polydimethylsiloxane (PDMS) casting. The splitting of microdroplets was inspected using an inverted microscope, and droplet production rates were measured using a high-speed camera. Furthermore, we verified that the microfluidic device could be used to prepare biodegradable chitosan microspheres.

## 2. Materials and Methods

### 2.1. Design of Microfluidic Device

**Figure 1:**
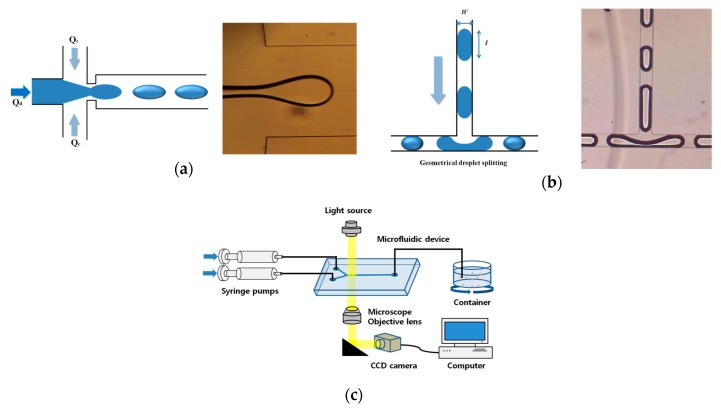
Schematics of (**a**) the flow-focusing part of the inlet and (**b**) droplet splitting at a T-junction in a microchannel in the 512-channel geometrical passive breakup device. (**c**) Experimental set-up for evaluating the microfluidic device (*Q*_d_: Dispersed phase; *Q*_c_: Continuous phase).

**Figure 2:**
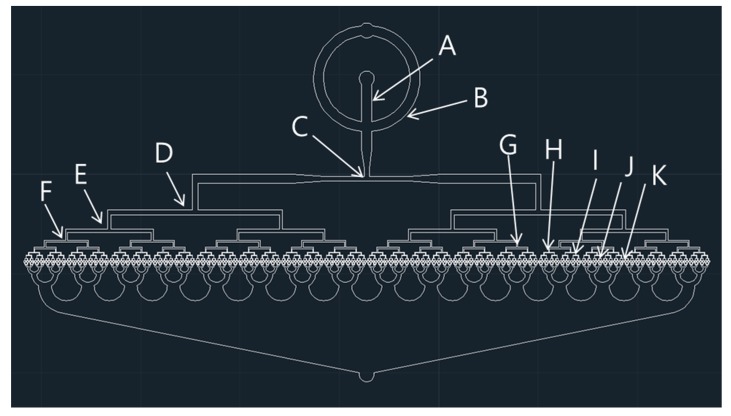
Computer-aided design (CAD) of the 512-channel geometrical passive breakup device.

Figure 1 shows the schematic of droplet splitting at a T-junction in a microchannel of the 512-channel geometrical passive breakup device. Firstly, an initial liquid plug is prepared at the flow-focusing part, as shown in Figure 1a. Then, the prepared liquid plug is split symmetrically into two at the T-junction (Figure 1b). The behavior of droplets in the microchannel is observed using a microfluidic system (Figure 1c). A total of 512 droplets with a narrow size distribution can be produced from a single droplet by splitting droplets at a series of T-junctions. The microfluidic device was designed using a computer-aided design (CAD) program (AutoCAD) (Autodesk, San Rafael, CA, USA). Figure 2 shows the design of the 512-channel geometrical passive breakup device.

As one channel is divided into two channels at the T-junction of a microchannel, after division at nine T-junctions, the number of channels at the final branch is 512. Thus, from a single microdroplet, at the final branch, 512 microdroplets are produced upon splitting nine times. Table 1 presents the specifications of the 512-channel geometrical passive breakup device. The width of the T-junction at the first inlet was chosen to be 1 mm to prepare a millimeter-sized micro liquid plug for droplet splitting (Figure 1a). The width of the microchannel was gradually decreased from 1000 µm to 30 µm. The droplet splitting can be expressed as follows:

(1) $$e=\frac{l}{\pi w}$$

where l is the length of a microdroplet before passing the T-junction, and *w* is the width of the channel at the T-junction (Figure 1b). As *e* increases, the minimum size of the droplet that can be split decreases at the T-junction. If *e* exceeds 0.95, droplets are divided at the T-junction regardless of the flow rate in the microchannel and the properties of the liquid [15]. Equation (1) indicates that, if the width of the microfluidic device is 30 μm, the length of a microdroplet, before passing the T-junction, should be longer than 90 μm to be divided at a T-junction. Therefore, for droplet splitting at the T-junction of the last branch, the area of the micro liquid plug should be larger than 2690 μm^2^. Because the production of 512 droplets requires nine instances of droplet splitting, the area of the initial liquid plug should be 1,375,200 μm^2^. If it is assumed that the liquid plug is circular, an initial liquid plug with a diameter of 420 μm should be prepared for stable microdroplet preparation. Therefore, the microchannel width in the flow-focusing part was set to be 400 μm to prepare an initial microdroplet larger than 400 μm in the squeezing mode and dripping mode. Furthermore, experimental results showed that the diameter of a microdroplet that can be divided is smaller than the theoretical diameter determined from the flow rate of the solution injected. The following equation describes the displacement of pressure in the microchannel:

(2) $$\Delta P=R_{h}Q$$

where $R_{h}$ and *Q* are the hydraulic resistance and flow rate in the microchannel. The displacement of pressure is proportional to the flow rate. $R_{h}$ can be expressed as follows:

(3) $$R_{h}\approx \frac{12µL}{wh^{3}\left(1-0.63(\frac{h}{w})\right)}\;\left(w>h\right)\;or\;\frac{12µL}{\left(1-0.917\times 0.63\right)h^{4}}\;\left(w\approx h\right)$$

where *µ* is the viscosity of the fluid, and *L* is the length of the microchannel through which the microdroplet passes; *w* and *h* are the width and height of the microchannel. The first version of Equation (3) is used when the width is larger than the height at the microchannel. Furthermore, we assume that the thickness and width of the microchannel at the eighth and ninth T-junctions are equal (w≈h). In this case, the second version Equation (3) can be considered to obtain $R_{h}$. Additionally, the $R_{h}$ values of the collection area and initial flow-focusing area were excluded.

In order to analyze the $R_{h}$ of the microchannel, the $R_{h}$ of the microchannel can be considered to be equal to the electrical resistance. When microchannels are connected in series, $R_{h}$ can be expressed as

(4) $$R_{h}=R_{h1}+R_{h2}$$

When the microchannels are connected in parallel, it can be expressed as

(5) $$\frac{1}{R_{h}}=\frac{1}{R_{h1}}+\frac{1}{R_{h2}}$$

The total $R_{h}$ of the microchannel, except for the orifice to prepare the initial liquid plug, can be obtained from Equation (5) as

(6) $$R_{h}=\overset{a}{\underset{n=1}{\sum }}\frac{R_{n}}{2^{n-1}}$$

where *n* denotes the number of branches of a T-junction in the microchannel about the $R_{h}$. If this equation is used for integrating a 512-channel geometrical passive breakup device, in which *n* is 10, the equation can be expressed as

(7) $$R_{h}=R_{1}+\frac{R_{2}}{2}+\frac{R_{3}}{4}\ldots \ldots \ldots \ldots \;\frac{R_{10}}{512}$$

In this equation, it is evident that the ratio of the fluid resistance at the initial stage to the total $R_{h}$ of the microchannel is considerably higher than the ratio for the other branches. Therefore, the design of the first-stage T-junction is extremely important for minimizing the $R_{h}$ in the microchannel. As the number of microchannels connected in parallel increases, the $R_{h}$ tends to decrease. For a given flow rate, if the width of the microchannel is increased to reduce the $R_{h}$, the minimum size that the microdroplet should have for splitting increases. A narrower microchannel results in smaller microdroplets being split. However, there is a limit to the flow rate of the injectable fluid because the $R_{h}$ increases with a higher flow rate. At the last branch of the microfluidic device, the two channels were connected to a common channel sequentially to prevent merging of microdroplets. The width of the common microchannel was doubled to maintain a constant flow rate so as to prevent droplet aggregation.

### 2.2. Fabrication Process for 512-Channel Geometrical Passive Breakup Device

The master mold of the 512-channel geometrical passive breakup device was fabricated using conventional photolithography with negative photo resist (SU-8 2100, MicroChem Corp., Newton, MA, USA). Firstly, a prepolymer of PDMS (Sylgard 184, Dow Corning, Midland, MI, USA) and a curing agent were mixed in the ratio of 10:1. The mixture solution was poured in the fabricated master mold and placed in an oven at a temperature of 90 °C for 2 h. After the curing process, the PDMS device was carefully separated from the master mold and bonded with slide glass. The assembled PDMS device was placed in the oven at 90 °C overnight to facilitate the recovery of its hydrophobicity.

### 2.3. Experimental Set-Up for Microfluidic Device

Deionized water was used as the dispersed phase, and 97 wt % mineral oil (M5904, Sigma Aldrich, St. Louis, MO, USA) and 3 wt % SPAN^®^80 (S6760, Sigma Aldrich, USA) were used as the continuous phase. SPAN^®^80 was used to prevent merging with each microdroplet in the microchannel before coming out from the device. The dispersed and continuous phases were injected into the fabricated microfluidic device using syringe pumps (Pump 11 Elite & Pico Plus, Harvard Apparatus, Holliston, MA, USA). Microdroplets in the microchannel were inspected using an optical inverted microscope (CSB-IH5, Samwon Scientific Ind. Co., Ltd., Seoul, Korea) as shown in Figure 1c. The diameter of microdroplets was analyzed using the Image J program with at least 30 samples. The microdroplet production rate was measured using a high-speed camera (Motionxtra N3, IDT, Tallahasse, FL, USA).

### 2.4. Preparation of Chitosan Microspheres with Fabricated Microfluidic Device

As the dispersed phase, a 1 wt % chitosan solution (5–19 kDa, 448869, Sigma Aldrich, USA) was prepared in a 1 wt % hydrochloric acid solution (DEAJUNG, Seoul, Korea). Mineral oil was prepared with 5 wt % SPAN^®^80 as the continuous phase. Mineral oil with 5 wt % glutaraldehyde (25% in H_2_O, G5882, Sigma Aldrich, USA) was prepared with 5 wt % SPAN^®^80 as the solidification solution. SPAN^®^80 was used to avoid merging with microdroplets before solidification of chitosan microspheres.

The microfluidic device was bonded with slide glass through plasma treatment, and the assembled microfluidic filter device was placed in the oven at 90 °C overnight for the PDMS to recover its hydrophobicity, which was necessary for the stable preparation of chitosan microspheres. The chitosan microspheres were prepared by injecting the prepared solutions into the microfluidic device with syringe pumps. The prepared chitosan microspheres were collected in the solidification solution after 5 h of stirring at 300 rpm. The solidified chitosan microspheres settled at the base upon centrifugation at 6000 rpm. The residual mineral oil was removed from the microspheres by washing them with acetone several times. The satellites of chitosan microspheres were removed using a sieve (10 μm). The collected chitosan microspheres were inspected using optical microscopy and SEM (scanning electron microscopy, JSM 6510, JEOL, Tokyo, Japan), and the diameter of chitosan microspheres was measured using the image J program.

## 3. Results and Discussion

As the width of the microchannel decreased, the minimum diameter that a droplet should have to be split at a T-junction decreased. However, the flow rate was restricted because the flow $R_{h}$ of the microchannel increased with the flow rate. Therefore, we attempted to minimize the hydraulic and split droplets stably at T-junctions by gradually reducing the width of the microchannel at each branch.

**Figure 3:**
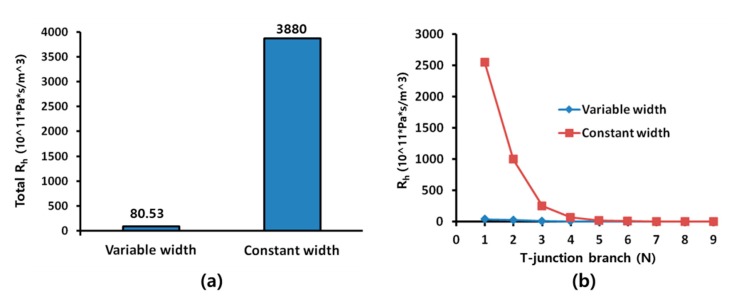
Comparison of the analytical $R_{h}$ values of a microchannel with variable width and the analytical $R_{h}$ values of a microchannel with a constant width at a T-junction branch: (**a**) total $R_{h}$ of T-junction branch; (**b**) $R_{h}$ at each T-junction.

Figure 3 shows an analytical comparison of the $R_{h}$ of the microchannel for a gradual decrease in the width from 1000 μm to 30 μm with that of a microchannel with a constant width of 30 μm. The total $R_{h}$ values of variable width and constant width were 80.53 × 10^11^ and 3880 × 10^11^ Pas/m^3^ (Figure 3a). The total $R_{h}$ of the microchannel with a gradually decreasing width decreased to 2.1% of that in the constant-width microchannel. Furthermore, the $R_{h}$ of the microchannel at each branch was evenly distributed after the improvement of the microchannel design (Figure 3b).

**Figure 4:**
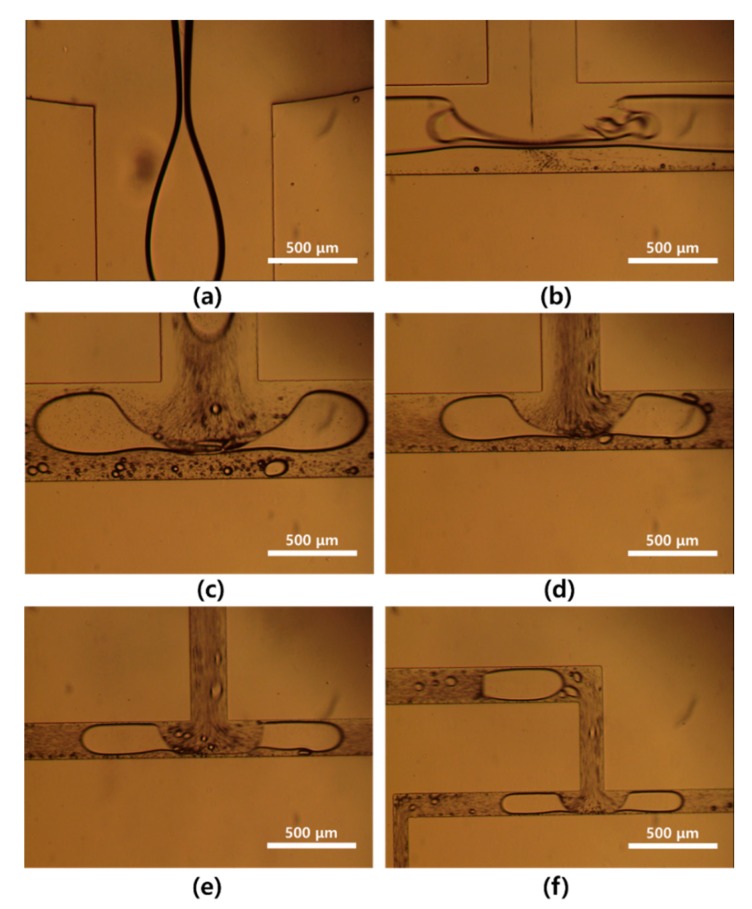
Optical images of (**a**) a water droplet at the orifice and (**b**–**f**) division of water droplets from the first T-junction to the fifth T-junction in the 512-channel geometrical passive breakup device.

**Figure 5:**
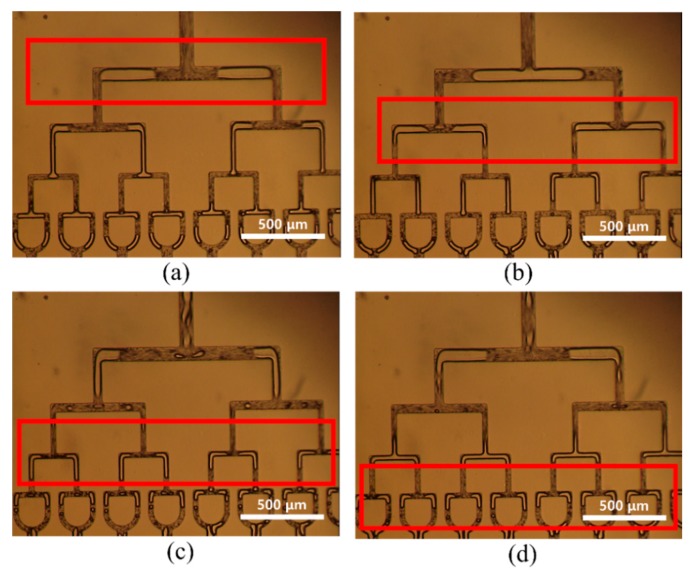
(**a**–**d**) Optical images of divided water droplets from the sixth T-junction to the ninth T-junction in the 512-channel geometrical passive breakup device.

Figure 4 shows optical images of droplet splitting from the first T-junction to the fifth T-junction. Relatively irregular microdroplets were produced from the first T-junction branch to the third T-junction branch because of the initial microdroplet size. Figure 5 shows optical images of the monodispersed microdroplets produced from the sixth T-junction to the ninth T-junction. These microdroplets had more stable shapes because of the reduced size of the microdroplets.

We observed that microdroplets were split stably at the final T-junction. The satellites (smaller than 1% of the parent droplet) were observed during droplet splitting at T-junction. The satellites were generated from the parent droplet as a result of the imbalance of capillary forces during the break-off of the droplet [17]. The formation of satellites was not influenced by the geometry of the microchannel but the viscosity ratio of solutions [18]. The satellites could be removed or significantly reduced at a viscosity ratio (viscosity of dispersed phase/viscosity of continuous phase > 1) greater than 1 [18]. However, in this study, the satellites were excluded in the size distribution because the satellites could be removed during the next process.

**Figure 6:**
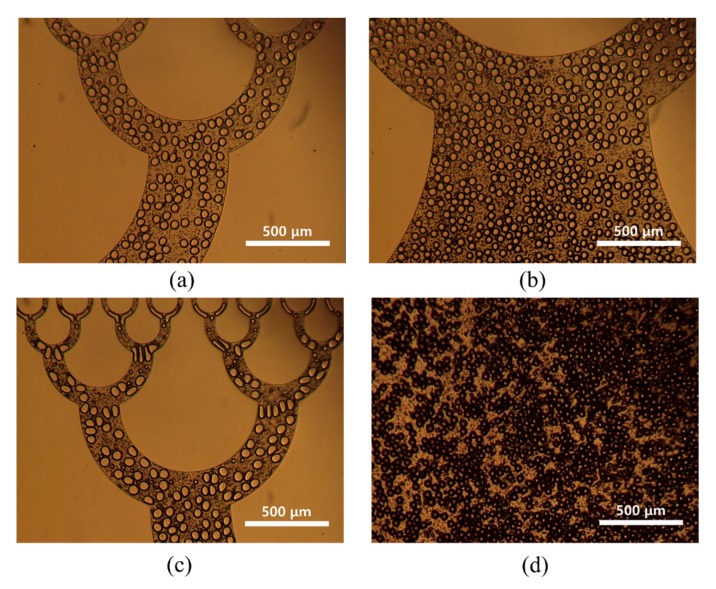
Optical images of prepared water droplets in the collection areas in the 512-channel geometrical passive breakup device: (**a**) first and second collection areas; (**b**) third collection area; (**c**) fourth collection area; (**d**) final collection area.

Figure 6 shows optical images of microdroplets in the different collection areas of the microfluidic device. The microdroplets produced by the microfluidic device were stably collected in the collection areas without any merging between microdroplets.

**Figure 7:**
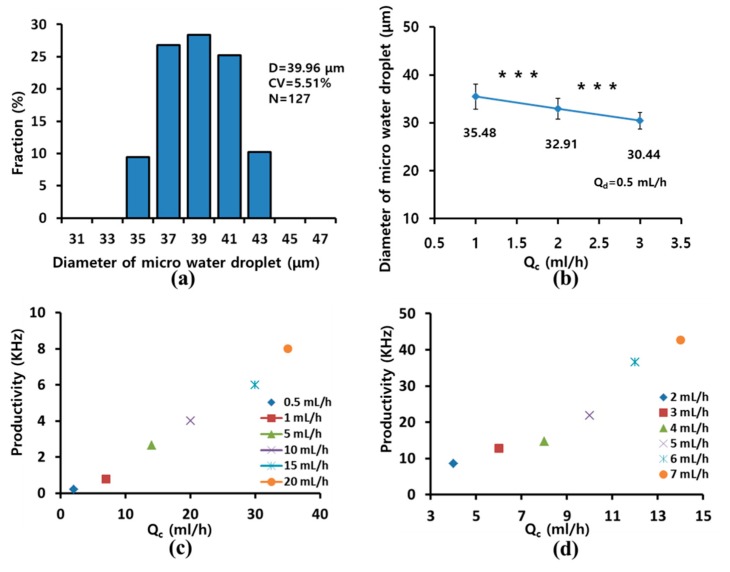
(**a**) Size distribution of water microdroplets produced by the 512-channel geometrical passive breakup device for *Q*_d_ and *Q*_c_ of 2 and 3 mL/h; (**b**) diameter of water microdroplets according to flow rate (*** *p* < 0.001); (**c**) productivity of microdroplets with 16-channel T-junction passive breakup; (**d**) productivity with 512-channel geometrical passive breakup device.

Figure 7a shows the size distribution of the microdroplets produced by the 512-channel geometrical passive breakup device. The diameter and coefficient of variation (CV) of the microdroplets were 40.0 ± 2.2 μm and 5.5% (*N* = 127) for *Q*_d_ and *Q*_c_ of 2 and 3 mL/h, respectively. Figure 7b shows the diameter of water microdroplets upon controlling flow rate. As *Q*_c_ increased with constant *Q*_d_, the diameter of water microdroplets decreased linearly. The diameter of water microdroplets decreased from 35.5 ± 2.6 μm (*Q*_c_ = 1 mL/h) to 30.4 ± 1.7 μm (*Q*_c_ = 3 mL/h). There were significant differences in diameter depending on *Q*_c_. The diameter of microdroplets slightly increased with increased the *Q*_d_, but there was no significant difference. On the other hand, the increase in production rate was more pronounced compare to the increase in diameter.

Figure 7c presents a graph describing the productivity of microdroplets in the 16-channel T-junction passive breakup device with various flow rates. Microdroplets were generated at 8 kHz for *Q*_d_ and *Q*_c_ of 20 and 30 mL/h. Figure 7d shows the productivity of microdroplets in the a 512-channel geometrical passive breakup device according to various flow rates. The production rate increased as *Q*_d_ and *Q*_c_ increased. Microdroplets were produced at 42.7 kHz by the microfluidic device for *Q*_d_ and *Q*_c_ values of 7 and 15 mL/h, respectively. The productivity of microdroplets of the 512-channel T-junction passive breakup device was 4.7 times higher than that of the 16-channel T-junction passive breakup device.

Table 2 is a summarized table for size distribution and droplet generation frequency [8]. Droplet production rates of the poly(methyl methacrylate) (PMMA) system (2.5 kHz) or silicone system (5.3 kHz) were higher than that of the PDMS system (0.1 kHz) due to their material strength. The productivity (42.7 kHz) of the 512-channel geometrical passive breakup device was significantly higher than their productivity.

Some groups reported higher productivity of microdroplets from their system than that of the 512-channel geometrical passive breakup device [12,13,14,19,20,21]. However, most researches required complicated fabrication processes or additional devices to resist the high pressure of the microchannel. Our approach does not require a complicated fabrication process or an additional device; it only uses a conventional PDMS casting process. The 512-channel geometrical passive breakup device can prepare microdroplets at 42.7 kHz without any additional device or treatment. Furthermore, because the presented approaches were based on droplet preparation from multiple nozzles, they considered the difference in *Q*_d_ due to the difference in flow resistance based on path length. The deference of the dispersed phase flow rate according to the position of the nozzle can cause a reduction in the monodispersity of microdroplets, and this phenomenon can be amplified by fabrication and assembly errors. In contrast to these approaches, the presented device is free from this problem because the presented method is based on geometrical droplet fission from a single droplet. Furthermore, in order to demonstrate the performance of the fabricated microfluidic device, we attempted to prepare chitosan microspheres.

**Figure 8:**
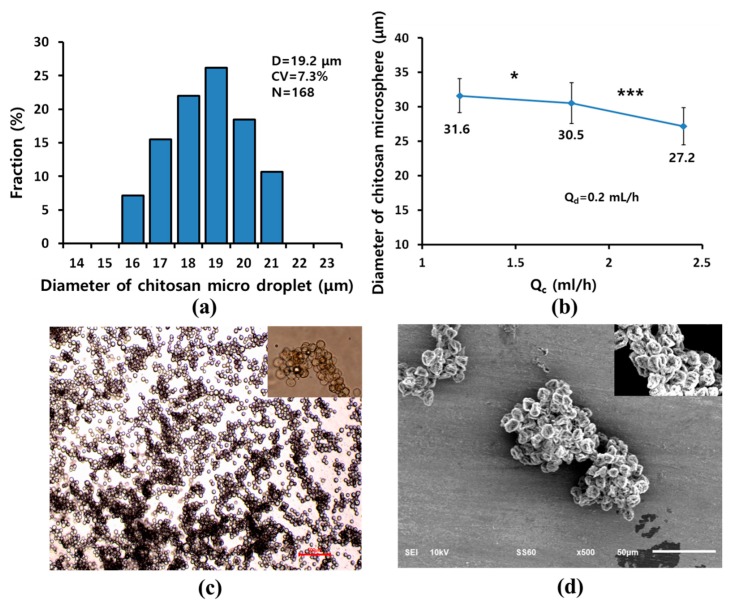
(**a**) Size distribution of chitosan microspheres produced using the 512-channel geometrical passive breakup device for *Q*_d_ and *Q*_c_ of 0.5 and 1.5 mL/h; (**b**) diameters of chitosan microspheres according to flow rate of various *Q*_c_ with constant *Q*_d_ (* *p* < 0.05, *** *p* < 0.001). The (**c**) optical and (**d**) SEM images of prepared chitosan microspheres.

Figure 8a shows the size distribution of chitosan microspheres prepared by the 512-channel geometrical passive breakup device with *Q*_d_ and *Q*_c_ of 0.5 and 1.5 mL/h. The diameter and CV of the prepared chitosan microspheres were 19.2 ± 1.4 μm and 7.3% (*N* = 168). The diameter of chitosan microspheres was smaller than the diameter of water microdroplets, as they had the same flow rates with increased polydispersity. The chitosan solution was formed as a stream at a flow rate ratio of water droplets because of higher viscosity. The diameter of chitosan significantly decreased as *Q*_c_ increased (Figure 8b). The diameter of chitosan microspheres decreased from 31.6 ± 2.5 μm (*Q*_c_ = 1.2 mL/h) to 27.2 ± 2.7 μm (*Q*_c_ = 2.4 mL/h).

Figure 8c is an optical image of the prepared chitosan microsphere. During the preparation of chitosan microspheres, satellites were generated upon splitting from the microsphere. The satellites were removed using a 10-μm sieve after the washing process. When the chitosan microspheres were inspected by SEM (Figure 8d), the microspheres were shrunk by water extraction. Nanometer wrinkles were observed on the surface of the chitosan microspheres. Recently, chitosan microspheres were considered as a tool to remove cell-free DNA using their inherent positive charge. As further research, we will prepare porous chitosan microsphere to enhance protein absorption as a DNA scavenger.

## 4. Conclusions

We presented a 512-channel geometrical passive breakup device for the production of microspheres. The microchannel of the microfluidic device was designed and modified using a CAD program, focusing on the minimization of hydraulic resistance and the stable preparation of microdroplets. The microfluidic device was fabricated using conventional photolithography and PDMS casting, and it was used for preparing water microdroplets at 42.7 kHz for *Q*_d_ and *Q*_c_ of 7 and 15 mL/h, respectively, to evaluate its performance. The diameter and CV of the prepared microdroplets were 40.0 ± 2.2 µm and 5.5% for *Q*_d_ and *Q*_c_ of 2 and 3 mL/h, respectively. The productivity of the microdroplets increased with *Q*_d_ and *Q*_c_. Furthermore, monodispersed chitosan microspheres were successfully prepared using the microfluidic device.

## Figures and Tables

**Table 1:** Specifications of the 512-channel geometrical passive breakup device.

Specifications	Width (μm)
A: Width of input A	1000
B: Width of input B	1000
C: Width of first T-junction	500
D: Width of second T-junction	540
E: Width of third T-junction	330
F: Width of fourth T-junction	200
G: Width of fifth T-junction	130
H: Width of sixth T-junction	80
I: Width of seventh T-junction	50
J: Width of eighth T-junction	30
K: Width of ninth T-junction	30
T: Thickness of the device	40

**Table 2:** Size and frequency of water droplets in oil in various microfluidic systems. PMMA—poly(methyl methacrylate); PDMS—polydimethylsiloxane.

	Geometry and Material	Continuous Phase	Droplet Size (μm)	Frequency (Hz)
**Water in oil**	Channel array, silicon	Kerosene with monolaurate	21	−5300 (est.)
	T-junction, acrylated urethane	Decane, tetradecane, and hexadecane with Span 80	10–350	20–80
	T-junction, PMMA	High-oleic sunflower oil	100–350	10–2500
	T-junction, PDMS	C_14_F_12_ with C_6_F_13_(CH_2_)_2_OH	7.5 nl	2
	Shear-focusing, PDMS	Oleic acid	13–35	15–100

## References

[1] Kshirsagar D.S., Saudagar R.B. (2016). Microsphere: A review. Res. J. Top. Cosmet. Sci..

[2] Sahil K., Akanksha M., Premjeet S., Bilandi A., Kapoor B. (2011). Microsphere: A review. Int. J. Res. Pharm. Chem..

[3] Kim C.M., Ullah A., Chang C.H., Kim G.M. (2017). Preparation of lidocaine-loaded porous Poly (lactic-co-glycolic acid) microparticles using microfluidic flow focusing and phosphate buffer solution porogen. Int. J. Precis. Eng. Manuf..

[4] Kim C.M., Ullah A., Kim K.G., Kim S.Y., Kim G.M. (2016). Preparation of carbon nanotube-wrapped porous microparticles using a microfluidic device. J. Nanosci. Nanotechnol..

[5] Berkland C., Kipper M.J., Narasimhan B., Kim K.K., Pack D.W. (2004). Microsphere size, precipitation kinetics and drug distribution control drug release from biodegradable polyanhydride microspheres. J. Control. Release.

[6] Xu Q., Hashimoto M., Dang T.T., Hoare T., Kohane D.S., Whitesides G.M., Langer R., Anderson D.G. (2009). Preparation of monodisperse biodegradable polymer microparticles using a microfluidic flow-focusing device for controlled drug delivery. Small.

[7] Whitesides G.M. (2006). The origins and the future of microfluidics. Nature.

[8] Teh S.-Y., Lin R., Hung L.-H., Lee A.P. (2008). Droplet microfluidics. Lab Chip.

[9] Squires T.M., Quake S.R. (2005). Microfluidics: Fluid physics at the nanoliter scale. Rev. Mod. Phys..

[10] Fujii T. (2002). PDMS-based microfluidic devices for biomedical applications. Microelectron. Eng..

[11] Kim C.M., Park S.J., Kim G.M. (2015). Applications of PLGA microcarriers prepared using geometrically passive breakup on microfluidic chip. Int. J. Precis. Eng. Manuf..

[12] Nisisako T., Torii T. (2008). Microfluidic large-scale integration on a chip for mass production of monodisperse droplets and particles. Lab Chip.

[13] Kobayashi I., Wada Y., Uemura K., Nakajima M. (2010). Microchannel emulsification for mass production of uniform fine droplets: Integration of microchannel arrays on a chip. Microfluid. Nanofluid..

[14] Fu G., Tor S.B., Loh N.H., Hardt D.E. (2010). Fabrication of robust tooling for mass production of polymeric microfluidic devices. J. Micromech. Microeng..

[15] Link D.R., Anna S.L., Weitz D.A., Stone H.A. (2004). Geometrically mediated breakup of drops in microfluidic devices. Phys. Rev. Lett..

[16] Amstad E., Datta S.S., Weitz D.A. (2014). The microfluidic post-array device: High throughput production of single emulsion drops. Lab Chip.

[17] Tan Y.C., Cristini V., Lee A.P. (2006). Monodispersed microfluidic droplet generation by shear focusing microfluidic device. Sens. Actuators B Chem..

[18] Derzsi L., Kasprzyk M., Plog J.P., Garstecki P. (2013). Flow focusing with viscoelastic liquids. Phys. Fluids.

[19] Jeong H.H., Yelleswarapu V.R., Yadavali S., Issadore D., Lee D. (2015). Kilo-scale droplet generation in three-dimensional monolithic elastomer device (3D MED). Lab Chip.

[20] Conchouso D., Castro D., Khan S.A., Foulds I.G. (2014). Three-dimensional parallelization of microfluidic droplet generators for a litre per hour volume production of single emulsions. Lab Chip.

[21] Kobayashi I., Neves M.A., Wada Y., Uemura K., Nakajima M. (2012). Large microchannel emulsification device for mass producing uniformly sized droplets on a liter per hour scale. Green Process. Synth..

